# A New Journal

**Published:** 1881-03

**Authors:** 


### A NEW JOURNAL.

The Microscope and its relation to Medicine and Pharmacy.

Chas. H. Stowell, M. D., Assistant Professor of Physiology and Histology, and Mrs. Louisa Reed Stowell, M. S., Assistant in Microscopical Botany, University of Michigan, Editors and Publishers.

This journal will be issued every alternate month, beginning April 15th, 1881.

The object of this publication will be to bring the microscope more prominently before physicians and pharmacists, and to show them how practically useful it may become in the hands of those who are far from being experts. It will especially deal with the adulterations of drugs and the means of detecting them; the normal and abnormal appearances of the secretions and excretions of the body; together with general hints and suggestions in regard to the manipulation of the instrument, the preparation of specimens, etc., etc.

It will also contain matter of interest to the general practitioner as reports will be given of the large clinics of our most prominent men.

This work will be as interesting and instructive to the dentist as to the physician. No dentist who desires to keep up with the progress of the times can afford to be without a good microscopic journal. Professor Stowell has made the histology of the teeth and the adjacent structures a special study, and hence will be able to present much that will profit the dentist.

The Microscopic Journal will be published at $1 per year.

Send for specimen number to the editor, Ann Arbor, Mich.

